# Bacteria-Assisted Transport of Nanomaterials to Improve Drug Delivery in Cancer Therapy

**DOI:** 10.3390/nano12020288

**Published:** 2022-01-17

**Authors:** Carla Jiménez-Jiménez, Víctor M. Moreno, María Vallet-Regí

**Affiliations:** 1 CIBER de Bioingeniería, Biomateriales y Nanomedicina, CIBER-BBN, 28040 Madrid, Spain; carlaj05@ucm.es; 2 Departamento de Química en Ciencias Farmacéuticas, Facultad de Farmacia, Universidad Complutense de Madrid, Instituto de Investigación Sanitaria, Hospital 12 de Octubre i+12, 28040 Madrid, Spain; victomor@ucm.es

**Keywords:** bacteria, nanoparticles, drug delivery, cancer, nanocarrier

## Abstract

Currently, the design of nanomaterials for the treatment of different pathologies is presenting a major impact on biomedical research. Thanks to this, nanoparticles represent a successful strategy for the delivery of high amounts of drugs for the treatment of cancer. Different nanosystems have been designed to combat this pathology. However, the poor penetration of these nanomaterials into the tumor tissue prevents the drug from entering the inner regions of the tumor. Some bacterial strains have self-propulsion and guiding capacity thanks to their flagella. They also have a preference to accumulate in certain tumor regions due to the presence of different chemo-attractants factors. Bioconjugation reactions allow the binding of nanoparticles in living systems, such as cells or bacteria, in a simple way. Therefore, bacteria are being used as a transport vehicle for nanoparticles, facilitating their penetration and the subsequent release of the drug inside the tumor. This review would summarize the literature on the anchoring methods of diverse nanosystems in bacteria and, interestingly, their advantages and possible applications in cancer therapy.

## 1. Introduction

From decades ago to the present day, antineoplastic therapy has been based on the administration of highly cytotoxic drugs to inhibit the proliferation or cause the death of cancer cells [1]. This strategy involved the development of a wide variety of cytotoxic agents with rather non-specific cell targets that are not restricted to malignant cells but are also common to most cell types. This lack of selectivity of chemotherapeutics together with the unspecific drug accumulation in highly vascularized organs such as liver, kidney, or lungs usually involves serious damage to healthy tissues or organs and to the patient’s immune system [2]. In addition, the poor diffusion of drugs into deep regions of tumor tissue and the early emergence of multi-drug resistance (MDR) mechanism severely limits the therapeutic efficacy of chemotherapy and is often associated with increased morbidity and mortality [3].

In 1989, the essential discovery of the enhanced permeability and retention (EPR) effect by Matsumura and Maeda [4] enabled the development of Nanomedicine. The EPR effect consists of the selective accumulation of nanoparticles (NPs) into tumoral tissues thanks to the presence of fenestrations in the chaotic cell wall of tumoral blood vessels. Nanomaterials present unique characteristics that made them useful tools as drug delivery systems, as they optimize drugs pharmacokinetics and increase drugs accumulation in tumors [5]. Nanoparticles can prolong circulation half-life and solubility of drugs by retaining them inside their porous or hollow structure.

From this date, a myriad of different types of nanoparticles for drug delivery have been developed, from pure inorganic systems as ceramic particles [6,7,8] or metallic [9] to organic ones like micelles [10], liposomes [11], polymersomes [12], or polymer nanocapsules [13], as well as hybrid nanodevices that combine both natures [14,15]. Among them, mesoporous silica nanoparticles [16] have raised enormous interest since Prof. Vallet-Regí and collaborators first reported in 2001 the suitability of mesoporous silica nanoparticles (MSNs) type MCM-41 as a drug delivery system [17]. Versatility of MSNs allows that various strategies using them [18,19,20] or biomaterials [21,22] have also been employed to fight bacterial infection and destroy biofilms [23]. Nevertheless, the accumulation and penetration of nanomaterials into deep regions of tumoral tissues is limited by some biological barriers [24]. The distribution of nanomaterials along the extracellular tumoral matrix is governed by extravasation capacity and diffusion limitations of nanoparticles. This fact leads them to accumulate mostly in the perivascular areas of the tumor rather than in the inner hypoxic regions, and thereby strongly limiting the therapeutic efficacy of nanomedicines.

Currently, thanks to the increased understanding of the human immune system, the spotlight in cancer therapy is focusing on the activation of the immune system as a potential alternative to chemotherapy. At this point, an old concept is emerging: the use of bacteria as an antitumoral therapy [25]. First reports on treating cancer with bacteria date back to 1863 [26]. Intrigued by precedent observations, W. Busch and colleagues studied that there was a correlation between a reduction in tumor growth and bacterial infections. They intentionally infected a cancer patient by placing him into a bed in which another patient had previously died due to *Streptococcus pyogenes* infection. The patient became infected, as expected, but the tumoral mass diminished because of the infection. However, the patient died due to the limited capacity to control bacterial infections at that time.

Later, in 1898, doctor Willian Coley and colleagues started to study this phenomenon in depth [27]. They realized that achieving a precise balance between infection control and therapeutic benefit was essential for a successful therapy. Their solution was to administer a mixture of heat-inactivated *Serratia marcescens* and *Streptococcus pyogenes* bacteria (cocktail currently known as “Coley’s toxin”) to many patients affected with unresectable soft tissue sarcomas [28,29]. The treatment was surprisingly very effective, and this Coley’s vaccine was later employed with more than one thousand cancer patients during his career. Coley also found that the common symptom associated with tumor shrinkage was a strong febrile reaction, given that patients whose fevers reached 38–40 °C had a superior five-year survival in comparison with those patients that suffered little or no fever during the treatment [30].

Even though bacteria-mediated tumor therapy (BMTT) remained under investigation after the death of Dr. Coley, it was long forgotten. Nevertheless, advances in biomedical research in recent decades have shown Coley’s principles to be valid and have demonstrated that many cancers are highly sensitive to an enhanced immune system as a result of immunostimulation [31]. BMTT potentiate immune response by T Lymphocytes CD8+ and CD4+ and Neutrophils recruitment, together with cytokines and chemokines, and release with no effect on the surrounding healthy tissue [32].

Considering that our enemy’s enemy could be our friend, the reason for using bacteria as anticancer agents is primarily provided by their inherent immunogenicity. However, we can also take advantage of their easy genetic manipulation and their high motion capacity. Facultative aerobic and anaerobic bacteria preferably colonize the deep and hypoxic areas of tumors. This feature of bacteria is, therefore, advantageous for their use as drugs nanocarriers. The preparation of nano-biohybrid systems consisting of nanoparticles linked to motile bacteria allow the delivery of high amounts of therapeutic agents to areas of the tumors that are unachievable with conventional chemotherapeutics or nanomedicines. In this Review, we will focus on the description of nanobiohybrid systems developed until the date, remarking on their usefulness in cancer therapy.

## 2. Bacteria

### 2.1. Definition

Bacteria are unicellular microorganisms that do not possess a nuclear membrane. They are the most abundant and diverse group of living organisms on the planet. They reproduce by binary fission and many of them multiply rapidly. Although some species of bacteria cause disease, most of them are harmless to the human body. Bacteria are also very metabolically active and can adapt to environments where many changes occur, as they can mutate spontaneously to enhance their adaptation. Therefore, they have importance in different fields of medicine and, especially in recent years, in cancer therapy [33].

**Figure 1:**
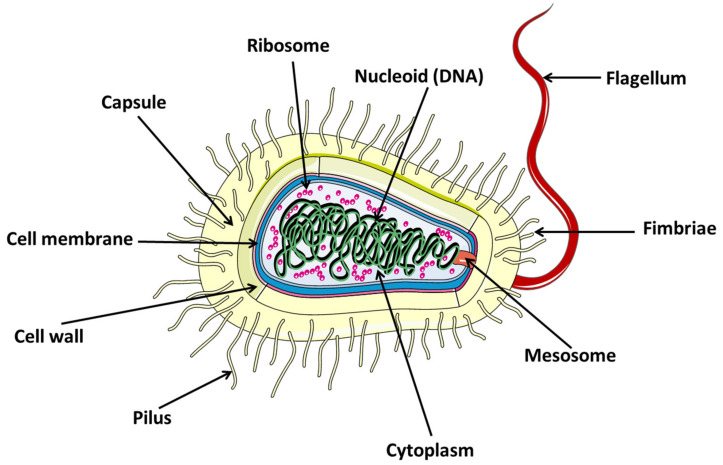
Structure of bacterial cell.

Bacteria present a very basic biological architecture, consisting of simple structures such as the cell wall, cell membrane, cytoplasm, nuclear material, plasmid, ribosomes, and others (Figure 1). On the other hand, there are also structures that are unique to some bacteria strains, such as the pilus, flagellum, spore, capsule, etc. (Figure 1).

### 2.2. Bacteria Types

Due to the diversity of existing bacteria, they can be classified according to their morphology [34], cell wall [35], or metabolism [33].

#### 2.2.1. Bacteria Based on Basic Shape

Bacteria have different shapes based on their cell bodies. For example, they can form rods (bacilli) of different helicities and curvatures, spheres (cocci), or rarer forms, such as stars, which are formed by polar growth. Moreover, they also show differences in their flagella or pili as they present different shapes, width, length, and the way they are positioned with respect to the cell body. In addition, their morphology can be changed in response to different environmental conditions or during their life cycle [36]. They can be simplified into three main groups according to their morphology:

- Cylindrical: These bacteria grow by increasing the length of the body cylinder. During their cell division, they are able to synthesize new cell poles for a short time, requiring thorough controls during cell division. In this group are *Bacillus subtilis, Corynebacterium diphtheria, Helicobacter pylori*, *Salmonella*, *Escherichia coli*, or *Caulobacter crescentus* (curved rods) [37].
- Coccal: This group grows through their division septa and must divide to grow. Their cell wall synthesis machinery is located in their division septa. In addition, because they depend on these septa and are spheroidal, they do not need to form chains, as in each generation their planes usually alternate. Thus, *Neisseria gonorrhoeae* divide into two alternating planes, *Deinococcus radiodurans* or *S. aureus* form bundles or clusters, and *Staphylococcus aureus* divide into three alternating planes [37].
- Ovococcal: Bacteria in this form usually grow through their dividing septum by modifying the extent of their length. They require changes in their mode of growth and need to place new dividing septa at the midpoint of the cell. If cell separation is not effective, these bacteria form cell chains. This group may include the bacterium *Streptococcus pneumonia* [37].

#### 2.2.2. Bacteria Based on Metabolism

From a biosynthetic point of view, bacteria can be classified into two main groups:

- Autotrophs: These bacteria have a very complex metabolism. They are capable of assimilating inorganic matter and transforming it into organic matter to produce the biomolecules necessary for their development. They are limited to using an inorganic source of carbon, such as CO_2_. These bacteria have no need to invade other organisms, nor do they need to break down dead organic matter to obtain the nutrients they need to survive [38].
  Depending on the metabolic system used by these bacteria to take inorganic compounds and transform them into organic compounds, they are divided into: Photoautotrophs (For the process of transformation of inorganic matter into organic matter, they use sunlight as a source of energy) and Chemoautotrophs (These bacteria need chemical energy to carry out their metabolic processes) [38]. Photoautotrophic bacteria can in turn be classified as oxygenic (they need photosynthesis to capture solar energy and convert it into chemical energy) and anoxygenic (these bacteria are anaerobic as they do not need oxygen for the respiration process) [39].
  Ultimately, these bacteria are important for ensuring the survival of other living things because they capture inorganic compounds that are toxic to other microorganisms. In addition, compounds released by these autotrophic bacteria can be assimilated by some heterotrophic bacteria.
- Heterotrophs: These bacteria use organic matter as a carbon source. This organic matter is transformed into energy and nutrients. Therefore, these organic materials are usually rich in energy, such as lipids, carbohydrates, and proteins. They need organic matter that has previously been synthetized by an autotrophic organism or other heterotrophic organisms. Other elements than carbon can be taken up as inorganic matter. Ultimately, some of these bacteria can cause infectious diseases in humans [40].

#### 2.2.3. Bacteria Based on Cell Wall

**Figure 2:**
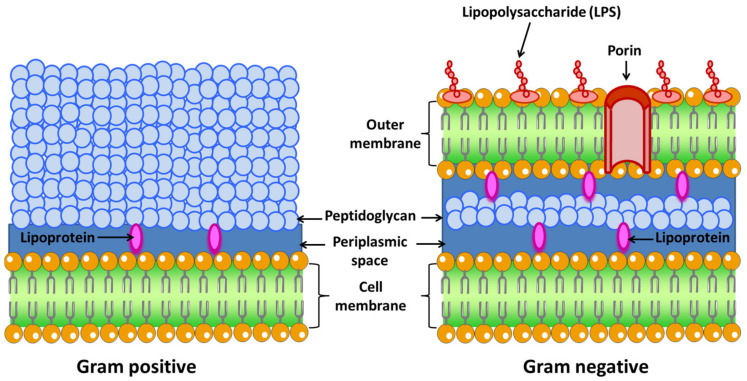
Differences between Gram-negative and Gram-positive bacteria.

In 1884, Christian Gram devised a staining procedure that enabled him to classify almost all bacteria into two major groups based on their cell wall (Figure 2) [41].

- Gram-negative: These bacteria possess a cell envelope that is composed of three main layers: the inner or cytoplasmic membrane, the peptidoglycan cell wall, and the outer membrane. The two membrane layers delimit a cellular compartment called the periplasm where a set of proteins are found [42]. From the outside to the inside, the outer membrane is the first layer. This membrane is characteristic of Gram-negative bacteria, while Gram-positive bacteria lack this organelle [43]. It is a lipid bilayer where phospholipids are found exclusively in the inner part of the membrane. The outer side is composed of glycolipids, mostly lipopolysaccharide (LPS). The human innate immune system is sensitized to LPS, an endotoxin that is recognized as antigen. It is a sure indicator of infection, since is responsible for the endotoxic shock associated with sepsis when caused by Gram-negative organisms [44].
  The proteins present in this membrane are usually classified into β-barrel proteins and lipoproteins. For example, in *E. coli*, the outer membrane contains few enzymes, but they are essential for ensuring their survival. These enzymes are a protease (OmpT) [45], an LPS-modifying enzyme (PagP) [46], and a phospholipase (PldA) [47]. The active site of these enzymes is oriented towards the outside of the cell (OmpT). The function of this membrane is to be a protective barrier. In fact, certain Gram-negative bacteria are more resistant to antibiotics than Gram-positive bacteria, i.e., *Pseudomonas*. In addition, LPS is central to the barrier function of this outer membrane, as it enables the maintenance and organization of this membrane. LPS is the most important surface antigen on these bacteria and therefore plays an important role in activating the immune system [48]. LPS is also responsible for Gram-negative bacteria-driven shock as it has an endotoxic action. Other functions include mediating adherence to cells and host tissues, inhibition of antibodies, and molecular mimicry [49].
  This membrane is bound to an underlying peptidoglycan by Braun’s lipoprotein (Lpp) [50]. Lipids attached to the amino terminus of this lipoprotein are embedded in the outer membrane. For example, Lpp is the most abundant protein in *E. coli*, with more than 500,000 molecules per cell [51]. This peptidoglycan is responsible for the rigidity of the bacterial cell wall and determines the morphology of the cell.
  Between the outer and inner membrane there is a watery cellular compartment called the periplasm, which is densely packed with proteins [52]. This compartment allows Gram-negative bacteria to capture potentially harmful enzymes, such as alkaline phosphatase or RNAase. It also contains periplasmic binding proteins important in chemotaxis, amino acid, and sugar transport, and chaperone-like molecules important in cell envelope biogenesis [53].
  As mentioned above, bacteria do not have intracellular organelles, so the membrane-associated functions of these organelles are performed in the inner membrane. Additionally, the proteins responsible for energy production, protein secretion, transport, and lipid biosynthesis are located in the inner membrane, although their location is different compared to eukaryotic cells [54]. This membrane is a lipid bilayer of phospholipids. For example, in *E. coli* phospholipids are found such as phosphatidyl glycerol, serine, and cardiolipin and phosphotidyl ethanolamine [55]. Within this group are *Escherichia coli*, *Salmonella*, *Hemophilus influenzae*, *Neisseria*, and *Bordetella pertussis*, among others.
- Gram-positive: The cell wall of Gram-positive bacteria is different from Gram-negative (Figure 2). First, they have no outer membrane. Lacking this membrane, the peptidoglycan layer is thicker than in Gram-negatives so that they can withstand the pressure exerted on the plasma membrane. They tend to live in harsh environments. Some of these bacteria are found in the gut. Anionic polymers called teichoic acids exist in the peptidoglycan layer, which are made up of repeated glycerol phosphates, ribitol phosphates, or glucosyl phosphates. These polymers make up 60% of the entire mass of the cell envelope of Gram-positive bacteria, making them responsible for the structure and function of the cell wall [56]. As there is no outer membrane to contain the extracellular proteins, these proteins have elements that cause them to be retained in the membrane or very close to it. Some of them are anchored to membrane-embedded lipids or have helices that pass through the membrane or bind or covalently associate with peptidoglycan [57]. This group includes *Streptococcus*, *Staphylococcus aureus*, *Clostridium botulinum*, and *Bacillus anthracis*, among others.

### 2.3. Peptidoglycans Biosynthesis

As mentioned above, there are two types of bacteria depending on their cell wall. Within them, there is a different synthesis and modification of peptidoglycans.

Peptidoglycan or murein is a macromolecule present in the bacterial cell. A peptidoglycan layer is formed around the cytoplasmic membrane [58]. In Gram-negative species, this structure is found in the periplasm between the outer and cytoplasmic membrane. However, this peptidoglycan layer is thicker and is connected to other cell wall polymers in Gram-positive bacteria. These polymers are the capsular polysaccharide, the S-layer, and the teichoic acid found in the wall [58]. The functions provided by this peptidoglycan are to maintain the shape of the bacterial cell, to maintain high turgor in the bacterium, and to provide rigidity to the flagella and pili which exert force to push or pull.

**Figure 3:**
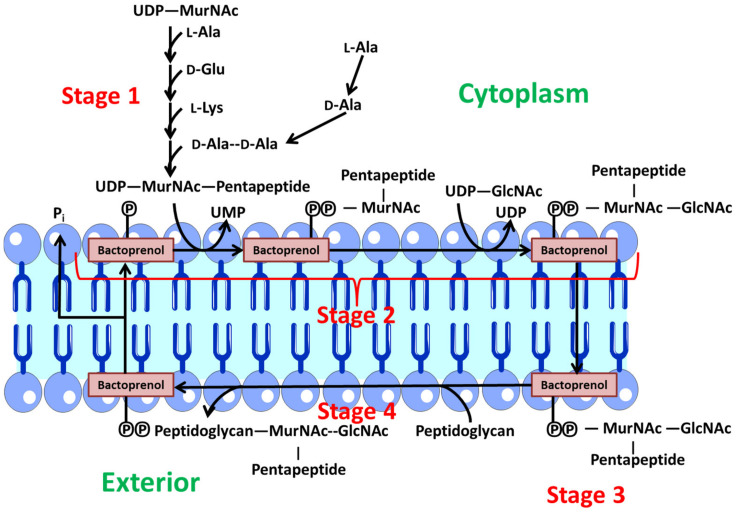
Peptidoglycans synthetic process.

This peptidoglycan layer is made up of polysaccharide chains composed of alternating *N*-acetylmuramic acid (MurNAc) and acetylglucosamine (GlcNAc) residues, which are connected by shorter peptides [58]. These peptides have d-amino acids such as d-glutamate and d-alanine. They can also be non-proteinogenic amino acids. Each species has different glycan chain length, cross-linkage structure, and amino acid sequence. These differences may occur at different stages of growth within the same species [59]. Chemical modifications are found in the peptide or glycan backbone [60]. It is necessary for this layer to remain intact in the bacterium, so the insertion and polymerization of new peptidoglycan strands is a difficult and regulated process [61]. This process consists of four stages (Figure 3):

- Stage 1: Synthesis of precursors in the cytoplasm. First, the monosaccharides *N*-acetylmuramic acid and acetylglucosamine, which form the peptidoglycan backbone, are activated by binding to uridine diphosphate. Then, a sequential and orderly addition of the various amino acids to *N*-acetylmuramic acid takes place. At this point, a pentapeptide is formed. Finally, the dipeptide d-alanyl-d-alanine binds. This dipeptide is synthesized in two steps. A first stage is through a racemase that converts l-ala to d-ala and a second stage where a peptide bond is formed between two d-ala [62].
- Stage 2: In this stage, these precursors are transferred to a lipid transporter (undecaprenyl-phosphate or bactoprenol (Lip-P)) in the cytoplasmic membrane, where the disaccharide units are created with the pentapeptide. At this point, a *Β* (1→4) bond is generated between MurNAc and GlcNAc. Therefore, Lip-P-P-MurNAc(pentapeptide)-GlcNAc is obtained. This polypeptide is anchored to the inner part of the membrane facing the cytoplasm via bactoprenol [62].
- Stage 3: Polymerization of various disaccharides. The bactoprenol is flipped from the inner to the outer layer, so that the precursor resulting from phase 2 is oriented towards the aqueous environment outside the membrane. At this point, polymerization of several disaccharide units takes place via a transglycosidation reaction. The Lip-P-P-MurNAc(pentapeptide)-GlcNAc disaccharide unit binds to the free end of another pre-existing chain, which is also bound to another Lip-P-P molecule. At this point, one of the Lip-P-P is released in its pyrophosphorylated form. An alkaline phosphatase acts on this molecule, which is responsible for eliminating the terminal phosphate, regenerating Lip-P again, which is then free to begin another cycle [62].
- Stage 4: The polymer generated in the previous stage is a linear chain of uncross-linked peptidoglycan bound to the membrane lipid transporter. This nascent polymer reacts with another pre-existing peptidoglycan acceptor via a transpeptidation reaction. The peptide bond generated between d-Ala (position 4) and d-Ala (position 5) of the nascent peptidoglycan is replaced by another peptide bond between the carboxylic group of the d-Ala (position 4) of the nascent peptidoglycan and the free amine group of the diamino acid (position 3) of the acceptor peptidoglycan. The energy for this reaction is provided by the hydrolysis of the peptide bond formed between the two terminal d-Ala, leading to the release of a d-Ala (position 5) in each transpeptidation reaction [62].

## 3. Bacteria in Cancer Therapy

### 3.1. Tumor Physiology

**Figure 4:**
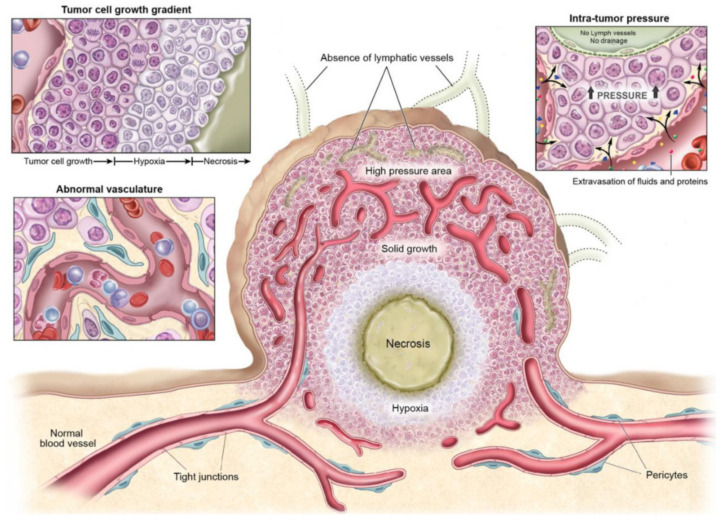
Physiological characteristics of a tumoral tissue. Reproduced with permission from [63]. Creative Commons (CC BY-NC) license from Theranostics, 2014.

Most solid tumors need nutrients and a regular supply of oxygen for their growth. The blood supply of the host organ performs these functions when tumors arise. However, due to their rapid growth, the vascular supply of the host is not sufficient to meet tumor needs at a certain point of time. Consequently, rapid cell proliferation conditions the physiology of the tissue (Figure 4), resulting in intrinsic characteristics of tumor tissue that are different from those present in healthy tissues [63].

- Abnormal vasculature: To meet the needs mentioned above, tumors develop their own functional vascular supply. For this, tumor cells secrete a series of pro-angiogenic factors that recruit endothelial cells for the formation of new blood vessels called angiogenesis [64]. The tumor vascular network formed is chaotic and irregular compared to the vascular supply of the normal tissue from which it begins to develop. This imbalance creates a neo-vasculature characterized by abrupt and leaky vessels that exhibit disordered branching and interconnection patterns. Ultimately, this vasculature is disordered and lacks a hierarchy of blood vessels compared to healthy tissues, where there is a regular and organized branching order [65]. These abnormalities cause a heterogeneity of tumor blood flow that interfere with the correct and homogeneous distribution of a drug within the tumor [66]. In addition, leaky blood vessels make it easier for macromolecules to reach tumor cells from the bloodstream, but also cause high interstitial pressures in tumors resulting in inhibition of drug accumulation in the tumor [67]. By this, an adverse microenvironment for cell growth is created within the tumor, which leads to the apparition of resistant cells to conventional cancer therapies as certain types of chemotherapy and radiation [68].
- High intratumoral pressure: Within tumors, tumor vessels do not supply blood efficiently due to high interstitial pressure favoring extravasation [69]. During proliferation, mechanical compression of the vessels together with high vascular permeability leads to increased interstitial fluid within the tumor. The interstitial hypertension exists due to the absence of lymphatic vessels preventing proper drainage of this extracellular fluid. In addition, this hypertension can inhibit drug diffusion and further compress the blood vessels by diverting blood from the center to the periphery of the tumor.
- High cell proliferation: Cell proliferation present different gradients due to the heterogeneity of the blood supply within the tumor microenvironment. This gradient means that cells close to the vessels increase rapidly while cells located in inner regions are deprived of nutrients. For this reason, the cell density is higher near the vessels compared to those far from the vessels. This increased cell density can also hinder drug penetration. On the other hand, hypoxic zones occur in the inner regions of the tumor that lack nutrients and oxygen supply, leading to the development of necrotic and senescent cells [70].

The tumor stroma is composed of the extracellular matrix, basement membrane, immune cells, fibroblasts, and vasculature [71]. Likewise, the array of cells that make up and inhabit the stroma is highly disparate, with cells such as cancer cells, tumor stem cells, inflammatory immune cells, pericytes, cancer-associated fibroblasts (CAFs), endothelial cells, and tumor stromal stem and progenitor cells, among many others. Therefore, to understand the biology of a tumor, it is necessary to consider both the cell types existing in the tumor and the “tumor microenvironment” generated during tumorigenesis [72].

The biological complexity of the tumor makes it difficult to select an appropriate treatment for patients. Although some of these tumor tissue characteristics are used to design effective and targeted therapies [73,74], many of them still complicate the proper dissemination of drugs and nanomedicines along the tumor.

### 3.2. Hypoxia as Chemoattractant for Bacteria

Among microenvironmental conditions that exist within the tumor, hypoxia (low oxygenation) has been the subject of most studies. The oxygen-deficiency of necrotic zones from tumors is a unique feature of solid tumors and is not present in healthy tissues [75]. Thus, this feature can be turned to advantage since it was noticed that hypoxic regions of tumors can be preferably colonized by some types of bacteria. Bacteria accumulation within tumors strongly depends on their tolerance to oxygen. Currently, three types of bacteria have been identified for BMTT, as tumor hypoxia serves as a chemoattractant for them [76]. Group I comprise obligate anaerobic bacteria, such as those of the genus *Bifidobacterium*; group II is composed of facultative anaerobic bacteria such as those belonging to the *Salmonella* and *Listeria* classes; and group III is composed of strictly anaerobic bacteria such as those of the *Clostridium* group.

For example, strict and obligate anaerobic bacteria such as Clostridium [77,78] and Bifidobacterium [79] do not tolerate oxygen, so they can only survive and proliferate in oxygen-deficient zones, which are found in the center of the tumor. The immune system is then activated and immune cells are attracted to the tumor to kill the bacteria [32]. Malmgren et al. tested this specificity by injecting Clostridium bacteria into mice bearing tumors. The results showed that only mice with tumors died from this infection [80]. Immune system activation is a key reason why bacteria can be excellent tools against cancer.

Another example is the use of facultative anaerobic bacteria as *Escherichia coli* [81], *Salmonella* [82,83], or *Listeria* [84,85], which can proliferate both in the presence and absence of oxygen. Five mechanisms are considered to control the accumulation of these bacteria in tumors: accumulation and preferential growth in tumor-specific microenvironments [86], accumulation in tumors when inflammation is present [87], chemotaxis of compounds produced by necrotic cells in tumor tissue (ribose, serine, and aspartate) [88], protection against eradication by the immune system [89].

Conventional antitumoral therapies present several drawbacks that hinder successful chemotherapy treatment, such as off-target accumulation, non-specific distribution, poor penetration into tumor tissues, and lack of selectivity. However, BMTTs have unique characteristics that overcome many of these shortcomings [90,91]. The main limitation of BMTTs is their pathogenicity, which can be overcome through genetic engineering by gene deletion [92,93]. This genetic modification has allowed the use of alive, non-pathogenic, attenuated, or genetically modified bacteria as an antitumor agent, providing the release of therapeutic biomolecules or direct tumor-killing effects. The selective replication and colonization of bacteria within the deep regions of tumors gives them a strong antitumoral effect as they are able to activate innate immunity and decrease side effects. In this case, the poor penetration of the drugs of conventional therapies in the hypoxic areas of the tumors mentioned above can be easily solved with a bacteria-based therapy. In addition, the presence of flagella in bacteria allows them to reach the innermost areas of the tumor tissue. Moreover, the expression of multiple ligands, cytokines, immunostimulants, and anti-tumor antigens can also be achieved by genetic manipulation of bacteria to enhance the therapeutic effect against specific tumors [72]. These unique features for cancer treatment are unattainable with both conventional chemotherapy and other immunotherapy-based therapies.

For example, *S. typhimurium* has been used in immunotherapies in murine trials where significant tumors shrinkage has been observed. These results have been made possible by local expression of bacteria or expression of immune-stimulating molecules in tumor cells, such as CCL21, IL-18, and Fas ligand [94]. Other preclinical studies have also applied *Bifidobacterium* bacteria in combination therapy with cytokines, such as granulocyte colony-stimulating factor (G-CSF), where increased anti-tumor effects have been observed [95].

On the one hand, the use of cancer vaccines, which aim to overcome the immune system’s tolerance to specific antigens unique to tumor cells, has attracted a great deal of interest in recent years. The function of this strategy is to target immune activity in a similar way to traditional vaccines, requiring the release of a vector expressing the desired gene. Bacteria that target immune-inducing cells are therefore good candidates for vaccine delivery [96].

## 4. Bacteria as Nanocarrier

### 4.1. Motion Capacity of Bacteria

There is another feature of bacteria that can be further exploited to enhance the therapeutic effect: their ability to move. In addition to producing antitumoral and immunostimulatory effects or being a recombinant factory of therapeutic agents, these motile bacteria can also serve as micro-swimmers for drug delivery within a living organism [97,98]. This cargo can be transported either linked to bacteria or loaded into a nanomaterial attached to bacteria. One of the main obstacles that greatly affect the therapeutic efficacy of nanomedicine-based therapies is the high interstitial fluid pressure (IFP) present in solid tumors as it becomes a barrier to transcapillary transport of nanoparticles. This elevated IFP, together with ineffective lymphatic drainage of tumor fluid, leads to pressure gradients towards the tumor margins [67]. As a result, inefficient extravasation of therapeutic agents towards the inner areas of the tumor occurs.

An adequate distribution of therapeutic agents or nanoparticles [99] within the neoplasia is mandatory to effectively affect the entire tumor cell population, but this scenario does not occur when particles accumulate in the perivascular regions of the tumor margins [100]. This fact generates tumor regions with low drug concentrations, which in turn favors the sprouting of quiescent cells that are largely unresponsive to chemotherapeutics. Consequently, it becomes mandatory that antitumoral therapy targets the deep and hypoxic areas of the tumor for an optimal treatment. This goal can be achieved by using a nanotransporter that allows direct drug delivery to these deep areas that are placed far from the tumor vasculature in order to reach the resistant cells located there.

BMTT enables successful intratumoral targeting due to the self-propulsion and guidance capabilities of bacteria. Guided by gradients of existing physiological conditions, such as hypoxia or nutrients, bacteria can actively swim away from the tumor vasculature and penetrate into deep regions where conditions are more favorable for bacteria growth. This inherent motility of flagellated bacteria allows them to penetrate tissues independently of hydrodynamic considerations [101]. In fact, bacteria can migrate and colonize distant areas from the tumor vasculature in greater proportion compared to the diffusion of drugs, the concentration of which strongly decreases as a function of distance from tumor vessels.

### 4.2. Distribution of Drugs inside Tumors

When non-pathogenic bacteria are injected systemically in BMTT, they can actively swim away along bloodstream. When bacteria reach the tumor vasculature, they can extravasated and preferentially accumulate within the hypoxic and nutrient-lacked deep areas of the tumor, thereby colonizing the regions where the necrotic and quiescent cells are placed [102]. By this way, the payload of the nanobiohybrid system is released, resulting in a significant drug accumulation at deep regions of the tumor for longer periods of time and at high concentration ratios if compared with simple drug diffusion from tumoral blood vessels. Released therapeutic (bio)molecules then diffuses from inward the tumor tissue to outward (where the blood vessels and well-fed cells are placed), in consonance with existing IFP gradient.

On the contrary, when chemotherapy or nanoparticles are systemically administrated, they extravasate by EPR effect and accumulate only in the regions proximal to blood vessels [103,104]. The existing high IFP avoids a correct efflux of drug from vessels, thus impairing the diffusion of molecules along the tumor. As a result, the deeper areas of the tumor remain deprived of drugs. Perivascular accumulation of passive molecules is undesirable as it leads to lower therapeutic efficacy in the deep zones of the tumoral tissue and generates more systemic toxicity.

The low perfusion in the tissue impairs drugs diffusion to deeper areas, resulting in a weak drug distribution throughout the tumor. Therefore, drug amounts at these regions are insufficient to induce apoptosis in tumoral cells, which favours the rise of drug-resistant cells. The surviving cells can undergo mutations and thus develop various MDR mechanisms, such as increasing P-glycoprotein efflux pumps on their membrane, reducing drug uptake membrane receptors, enhancing DNA-reparation mechanisms, or overexpressing enzymes responsible for drug inactivation, among others [105].

By contrast, the inversion of drug location achieved with BMTT affects tumors from the inside out, resulting in an enhanced cytotoxic effect in the deep regions of the tumoral tissue, where resistant cells are found. This strategy would have the similar effect of increasing therapeutic efficacy and decreasing systemic damage to normal tissues. Moreover, motion capacity of bacteria is particularly relevant. The transport of high amounts of drugs inside nanoparticles in conjunction with other therapeutic features of bacteria (as their immunogenicity and their facility to be genetically engineered) allows the creation of synergistic effects that can greatly improve the therapeutic outcome.

### 4.3. Bioconjugation in Living Organisms

The attachment of a nanomaterial to the surface of bacteria can be achieved by different strategies. The first one is simply by establishing electrostatic interactions between bacteria and nanoparticles [106,107,108]. This method involves the existence of a different net electrostatic charge between nanoparticles and the surface of bacteria. Bacterial outer envelope presents net negative electrostatic charge due to the existence of ionized phosphoryl and carboxylate groups, thus nanoparticles need to be positively charged to interact effectively.

Other reported approaches are the immobilization using acid-labile linkers [109] or by establishing biotin-based bioaffinity interactions, such as avidin-streptavidin or avidin-neutravidin ligations [110,111]. Although biotin methods present high affinity between the parts to be joined, the main limitation is the low coupling efficiencies achieved. Another strategy can be the formation of covalent bonds between nanoparticles and the surface of bacteria [112]. Additionally, nanoparticles can also be carried inside bacteria, being inserted by incubation and electroporation method [113].

Among these bioconjugation methods, the formation of covalent bonds has attracted great interest thanks to the innumerable chemical reactions that exist. Chemical reactivity allows endless combinations between two chemical groups that react bio-orthogonally. Bio-orthogonal reactions are those whose chemical groups react with each other in a rapid and chemoselective manner under physiological conditions (37 °C and pH of 6 to 8). The main characteristic of these reactions is that they form specific covalent bonds without interfering with the diversity of chemical functionalities involved in the biological processes of living systems.

**Figure 5:**
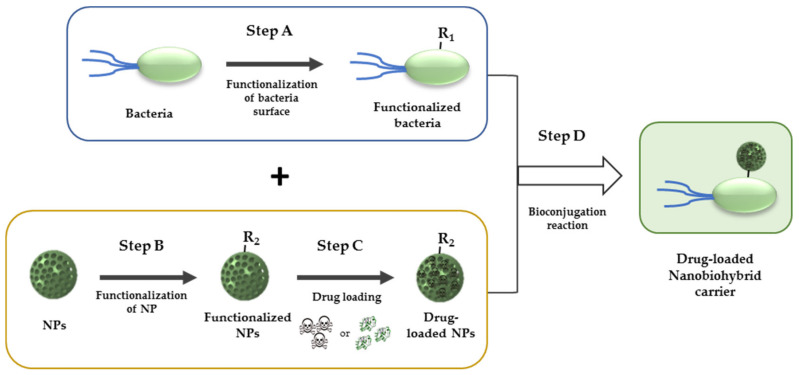
General scheme of bioconjugation reactions for the obtention of a drug-loaded nanobiohybrid carrier. Step A: First, a chemical group R_1_ is incorporated in the surface of bacteria. Step B: Nanoparticles are functionalized with the chemical partner of R_1_ (R_2_). Step C: Then, NPs are loaded with the desired therapeutic agent. Step D: Bioconjugation reaction between the chemical partners R_1_ and R_2_ leads to the bioconjugate bacteria-nanomaterial.

The field of chemical biology continuously develops bioorthogonal chemical reactions of interest for the conjugation of small molecules, nanomaterials, or biomolecules (such as proteins, glycans, lipids, or nucleic acids) with living organisms as cells or bacteria [114,115,116,117,118]. Bioconjugation reactions present enormous biological interest. Some of their possible applications are the labelling of living systems for sensing or imaging [119,120,121], or the incorporation of targeting elements for drug delivery and therapy [117,122,123]. Bio-orthogonal approach applied to living organisms consists in the incorporation of a unique chemical functionality onto the surface of the entities aimed to be linked together (Figure 5). These bio-orthogonal functional groups can be introduced in living systems in a non-perturbing manner, allowing thus their highly selective conjugation with the desired entity (i.e., drug, nanoparticle, or probe) through their incubation for a period of time.

For the covalent bioconjugation of nanoparticles [124] to the bacterial surface [125,126], there are several approaches. In general, one of the bio-orthogonal groups (R_1_) must be present or introduced in the surface of bacteria and should not (or minimally) perturb its biological function (Figure 5, Step A). On the other hand, NPs are functionalized on their surface with the chemical partner R_2_ (Figure 5, Step B) and then are loaded with the desired therapeutic agent if necessary (Figure 5, Step C). Thereafter, living organism is subsequently incubated with the nanomaterial (Figure 5, Step D) that has been previously functionalized with the R_1_-complementary chemical group (R_2_). The surface of nanoparticles can be easily functionalized with a wide variety of chemical functionalities [124,127], so this issue is not tough. Bio-orthogonal chemical reactions occur simply and efficiently to form the bacteria-nanomaterial hybrid conjugate.

A common strategy is the straightforward reaction of chemical groups that are naturally present in the surface of bacteria cell wall, such as amine or thiol groups, with their correspondent bio-orthogonal groups incorporated in the nanomaterial. Amines and thiols are moieties present in lateral chains of lysine and cysteine amino acids [117], which are frequently present in peptides and peptidoglycans founded in bacteria surface. They possess nucleophilic character, so the conjugation is possible if the chemical partner contains an electrophilic group. *N*-hydroxysuccinimide (NHS) esters (A in Table 1) and iso(thio)cyanates (B in Table 1) present an electrophilic carbonyl that reacts with amines to lead the correspondent amide or (thio)urea derivates, respectively.

Thiols from cysteine residues also present nucleophilic capacity and are commonly used for bioconjugation with maleimide (C in Table 1) or iodoacetamide (D in Table 1) groups, since the formation of the corresponding thioether derivate occurs rapidly and efficiently [117]. The other approach is the use of ketones or aldehyde groups that can be present in proteins or glycans for the formation of Schiff bases with primary amine groups [114,117]. The formation of a stable imine confers covalent character, but sensitivity to changes in pH made this bond reversible. Hydrazides (E in Table 1), aminooxy groups (F in Table 1), and primary amines (G in Table 1) react with carbonyls to form their corresponding hydrazone, oxime, and imine derivates. These chemical functionalities are very useful for the bioconjugation of living systems, but they lack bio-orthogonality. This means that these reactions are not very selective, since chemical groups involved are not chemically specific: they are found in most biological processes that sustain life.

To improve this poor chemo-specificity, an interesting strategy is the use of non-natural chemical entities (absent from all animal species) that specifically react with each other in aqueous media, regardless of the presence of other reactive chemical functionalities. Azide groups are suitable for this purpose, since they are stable at physiological conditions, apparently do not react with water, and are resistant to oxidation. Moreover, azides are mild electrophiles that do not react with nucleophiles present in biological systems such as amines. They exhibit excellent orthogonality with activated phosphines or alkynes, thus yielding readily [3+2] cycloadditions to form stable triazoles (H–J in Table 1). These types of bio-orthogonal chemical reaction are also known as “Click reaction”. Depending on the chemical partner employed, they can be classified as:

- Copper-catalyzed [3+2] azide-alkyne cycloaddition: Azides are 1,3-dipoles, thus can undergo reactions with dipolarophiles as activated alkynes (H in Table 1). The reaction is thermodynamically favourable since the dipolarophile is activated but requires Cu (I) catalyst for an efficient reaction [115]. However, present a major disadvantage since the metal catalyst exhibit cellular toxicity to bacteria.
- Strain-promoted [3+2] azide-alkyne cycloaddition: Represent a catalyst-free alternative that employ as complementary group a highly strained cyclooctyne ring (I in Table 1). The reaction between azide and strained alkyne is thermodynamically favoured at room temperature and no toxic effects are observable [114].
- Staudinger ligation: This transformation occurs between the nucleophilic phosphorous of triarylphosphine partner and the electrophilic nitrogen atom of azide, affording the amide-phosphine oxide derivate (J in Table 1) [115]. Reaction takes place at physiological pH with no apparent toxic effects.

Due to the non-natural origin of azides, they have to be incorporated de novo on the surface of bacteria using their own metabolic biosynthesis machinery [114]. As described in previous sections, the bacterial cell wall structure is composed by a rigid structure of peptidoglycans which, in turn, is composed of glycan strands cross-linked by short peptides of d-amino acids. Given the role of the biosynthetic mechanisms of bacteria in the incorporation of natural d-amino acids in peptidoglycans from diverse bacteria, researchers have exploited this mechanism for the incorporation of non-natural d-amino acids in peptidoglycans from bacterial surface [128]. On the other hand, the strain cycloalkyne is easily incorporated in the surface of desired nanoparticle. The strain-promoted azide-alkyne cycloaddition is thus straightforwardly accomplished with no Cu (I) catalyst required, and obtains good yields in physiological conditions.

The choice of the most appropriate bioconjugation reaction must be carefully studied depending on the bond desired in the nanobiohybrid system. Every reaction presents specific reactivity in term of chemoselectivity or performance [120,125,129,130], influenced by physicochemical factors. Although the most important chemical reporters are summarized in Table 1**,** the field is constantly growing [131], and new bioorthogonal ligations [132] or even cleavable bioorthogonal linkers [133] are also being developed.

## 5. Nanobiohybrid Bacterial Carriers

Since BMTT has emerged as a new strategy to effectively combat cancer, diverse bio-hybrid nanocarriers composed by a broad variety of nanomaterials have been reported. These nanocarriers have been employed for the delivery of therapeutic drugs, proteins, or genes to deep tumor regions that are unreachable by conventional chemotherapy, and the strategy used to combat cancer differs in each case from the nature of the nanomaterial. In addition, it has been studied that the anchoring of nanoparticles (NPs) on the surface of bacteria does not significantly affect their motility and viability [113,134,135], which makes them useful tools.

One of the first examples of bacteria-mediated transport of nanoparticles for cargo delivery as a model for a promising strategy for cancer therapy was reported in 2007 by Akin et al. They conjugated polystyrene nanoparticles onto *L. monocytogenes* surface by employing the high affinity avidin to streptavidin/neutravidin interaction [97]. For this, they used the biotinylated monoclonal antibody C11E9 that recognize a surface protein (*N*-acetylmuramidase) from bacteria, which subsequently were attached to streptavidin-labelled polystyrene nanoparticles. Then, a biotinylated DNA-plasmid encoding green fluorescence protein (GFP) was conjugated to the remaining streptavidin moieties from the surface of nanoparticles. Once they were internalized by the cells, plasmids were released from the nanoparticles and genes were expressed inside the cells. The efficient gene delivery and protein expression was evaluated observing GFP fluorescence in mice.

Another example of bacteria-mediated transport of drugs to the tumor is the work reported by Xie et al. [136]. They constructed a drug-carried bacterial nano-swimmer by attaching doxorubicin (DOX) directly to *E. coli* Nissle 1917 (EcN) using the pH labile cis-aconitic anhydride linker [137]. Despite conjugating DOX to bacteria, they observed that EcN viability remained over 70%. Moreover, EcN-ca-DOX accumulated in the tumor and slowly released the drug in a controlled and acid-responsive manner. Significant antitumor efficacy was observed for the nanosystem in 4T1 tumor-bearing mice. EcN-ca-Dox prolonged the animal survival, inhibited the tumor growth, and induced apoptosis in tumoral cells. Some other examples are described below, classified depending on the nanomaterial used in each case for their conjugation with bacteria.

### 5.1. Polymeric Nanoparticles

Hu, Wu, and colleagues designed a new strategy to deliver oral DNA vaccines for efficacious cancer immunotherapy [106]. In this case, they prepared DNA-loaded cationic polyethylenimine (PEI) nanoparticles, which were then used for coating attenuated *Salmonella* via electrostatic affinity interactions. This protective coating allowed bacteria to effectively escape phagosomes when administered orally. DNA sequence loaded in nanoparticles encoded vascular endothelial growth factor receptor 2 (VEGFR-2). Nanosystem showed a remarkable T cell activation, angiogenesis suppression in the tumor vasculature, and tumor necrosis.

Xie et al. employed a bio-orthogonal tetrazine/norbornene click reaction to conjugate pro-micelle drug-conjugated copolymers on *E. coli* Nissle 1917 to form EcN-PM_D/T_ nanosystem [109]. Amphiphilic copolymers possess acid-labile linkers that degrade under the mild-acidic pH present in tumors in a pH-responsive manner. By this, copolymers are release from bacteria and subsequently undergo self-assembling into micelles. A synergistic antitumor effect was achieved due to the release of both doxorubicin and *α*-tocopheryl succinate from self-assembled micelles once they are endocyted into tumoral cells.

Suh et al. described a nanocarrier (called NanoBEADS) where poly(lactic-co-glycolic acid) (PLGA) nanoparticles were conjugated with *Salmonella* thanks to the use of streptavidin–biotin high affinity interaction [110]. The effect of nanoparticle anchoring was evaluated testing the invasion capacity of NanoBEADS in 3D tumor spheroids in vitro, as well as their biodistribution in vivo in a tumor model. They observed that nanoparticle conjugation did not affect the capacity of bacteria to move towards intratumoral regions. NanoBEADS autonomous nanosystem enhanced the distribution and retention of nanoparticles in solid tumors by up to a remarkable 100-fold without requiring any externally applied driving force.

Other interesting nanocarrier is the developed by Xing and Yin et al. They used *Magnetospirillum magneticum* (AMB-1) bacteria that is characterized by containing magnetosomes inside the body that are susceptible to external magnetic fields [138]. Bacteria were conjugated to light-triggered indocyanine green PLGA nanoparticles (INPs) that were modified with maleimide groups in their surface. Covalent anchoring was obtained by Michael addition reaction of activated sulfhydryl from the surface of AMB-1 bacteria with maleimide on nanoparticles [139]. Tumor ablation was achieved by photothermal therapy using an on-demand near-infrared laser to activate the indocyanine green (ICG) photosensitizer of INPs. Thanks to the hypoxia targeting and magnetotactic motility of bacteria, they were autonomously directed and accumulated in the tumor. After laser irradiation, the biohybrid nanosystem induced a maximum temperature up to 58 °C, resulting in a successful tumor ablation.

*Salmonella* was also used by Chen and co-workers for hypoxia targeting of tumors [140]. Attenuated bacteria were conjugated to polydopamine nanoparticles via oxidation and self-polymerization, and the nanocarrier was then injected into tumor-bearing mice. The nanobiohybrid system effectively targeted hypoxic areas of the solid tumors, and once there, near-infrared laser irradiation of the tumors produced photothermal therapy by heating polydopamine nanoparticles. This combined therapy eliminated the tumor without relapse or metastasis with only one injection and laser irradiation. Another sophisticated nanosystem based in *E. coli* attached to DOX-loaded polyelectrolyte multilayer microparticles with incrusted magnetic nanoparticles also demonstrated effective and enhanced targeted drug delivery under magnetic guidance in vitro [141].

### 5.2. Silica Nanoparticles (SiNPs)

MSNs present unique characteristics to serve as drug delivery systems in cancer therapy, since they allow loading of high amounts of therapeutic agents into their pores [16,142,143,144]. In addition, MSNs present good biocompatibility [145,146,147] and their surface can be decorated with a wide range of chemical functionalities [123,127,148]. For this, Moreno, Álvarez et al. designed a bio-hybrid nanocarrier based on the attachment of MSNs, which are loaded with Doxorubicin, over the surface of *E. coli* bacteria [112]. In this work, the covalent attachment of MSN to bacteria was achieved with the employment of a cooper-free strain-promoted azide-alkyne cycloaddition reaction, also known as “click reaction”, which is compatible with living systems. On one hand, MSN were functionalized with dibenzylcyclooctyne (DBCO) groups. On the other hand, bacteria were fed with azide-d-alanine amino acid, which was metabolized and incorporated on bacteria cell wall. By this strategy, they achieved a successful transport of the drug-loaded nanoparticles to inner regions of a 3D collagen gel. The biohybrid nanocarrier was evaluated in a 3D tumoral tissue model and was tested to produce a double therapeutic effect. The nanosystem provoked a decrease of around 80% in viability of HT1080 human fibrosarcoma tumoral cells, while it was also able to provoke an immunogenic response.

Another interesting application of silica nanoparticles is the nanocarrier described by Tang and colleagues, which was designed for photodynamic ablation and ultrasensitive imaging of bacteria for infection treatment [149]. They prepared a nanomaterial composed of green-fluorescent silica NPs functionalized with a gluco-polymer and loaded with Ce6, which was internalized by bacteria via interaction of glucosyl polymers with the specific adenosine triphosphate (ATP)-binding cassette (ABC) transporters present on the bacterial cell membrane. Precise imaging was obtained by tracking the red fluorescence of Ce6 and the green fluorescence related to SiNPs, allowing in vivo detection of bacteria as low as 10^5^ colony-forming units. Moreover, Ce6 served as a photosensitizer that generates reactive oxygen species (ROS) under 40 min of low power laser irradiation at 600 nm [150] to kill bacterial cells. The nanosystem exhibited photodynamic antibacterial efficiencies of 98% against *S. aureus* and 96% against *P. aeruginosa.* This strategy could also be applicable for cancer therapy, employing bacteria as nanocarriers.

### 5.3. Carbon Nanoparticles

An interesting anticancer therapy involves the in situ generation of cytotoxic species by photoinduced reduction of NO_3_^−^ to NO. Zheng and co-workers have developed a nanosystem for photo-controlled bacterial metabolite therapy [107] by anchoring bacteria with a nano-photocatalyst that enhance their metabolic activities. Carbon nitride (C_3_N_4_) doped with carbon dots was combined with *E. coli*, which possess enzymes that catalyse the reduction of reduction of endogenous NO_3_^−^ to cytotoxic NO. Under light irradiation, photoelectrons produced by C_3_N_4_ are transferred to *E. coli* to promote the enzymatic reduction, obtaining a 37-fold increase in NO intratumoral concentrations. In a mouse model, C_3_N_4_-loaded accumulated perfectly throughout the tumor, and phototherapy treatment resulted in about 80% inhibition of tumor growth.

### 5.4. Metallic Nanoparticles

One case employing Au NPs is the work reported by Park and co-workers involving branched Au NPs that are conjugated to polyethyleneimine (PEI)-decorated *Clostridium novyi*-NT spores through formation of electrostatic interactions [151]. In this case, they made use of gold nanoparticles for computed tomography image monitorization of nanocarrier movement. The bacteriolytic capacity was evaluated in a PC3 human prostate tumor xenograft mouse model, showing that AuNP-coated *C. novyi*-NT spores have the potential to serve as a delivery platform for a combination tumor therapy.

Other example that employ Au NPs is the nanocarrier developed by Luo et al. for tumor hypoxia targeting [152]. They made use of two different nanoparticles delivery strategies involving a cargo-carrying method and an antibody-directed method. For this, anaerobic *Bifidobacterium breve* and *Clostridium difficile* spores were conjugated by electrostatic interactions and antibody affinity, respectively, with upconversion nanorods for imaging and Au nanorods for photothermal ablation of tumor upon near-infrared light excitation. The antibody-directed strategy showed the most effective treatment, resulting in longer bacteria retention and effective therapy removing completely tumors.

Dong et al. have reported the conjugation of citrate-stabilized Au nanoparticles (NPs) to the surface of *E. coli* bacteria through a novel conjugation method [153]. Bacteria were engineered to incorporate an inducible gene that regulates the display of peptides with desired sequences in the surface of bacteria. These peptides contain known gold-binding peptide sequences that allow the conjugation of nanoparticles by metal-peptide affinity. Their experiments demonstrated a successful loading of Au NPs with a correlation in binding affinity as a function of the specific peptide engaged, which show potential applications in cancer therapy.

Magnetic Fe_3_O_4_ nanoparticles were used by Fan, Peng, and co-workers for tumor therapy due to in situ generation of H_2_O_2_ and subsequent generation of toxic hydroxyl radicals via Fenton-like reaction [154]. For this, they employed an engineered *E. coli* MG1655 designed to overexpress NDH-2 enzyme, which is responsible for localized H_2_O_2_ generation in the tumor. Fe_3_O_4_ NPs were covalently linked to bacteria through amide condensation to act as a catalyst for Fenton-like reaction. This reaction converts H_2_O_2_ to hydroxyl radicals that induce tumor cell apoptosis. This nanosystem achieved an effective tumor colonization and self-supplied therapeutic effect in a CT 26 tumor bearing Balb/c mice model.

### 5.5. Liposomal Nanoparticles

Similarly to MSNs, liposomes offer many advantages as drug delivery systems [155]. They provide biodegradability, biocompatibility, and the ability to encapsulate high number of drugs, proteins, genes, or other molecules, depending on their hydrophilic or hydrophobic nature. Moreover, their size and surface groups can be easily tuned, which makes them a very employable system for cancer treatment.

Zoaby and co-workers prepared a biohybrid nanosystem consisting on liposome-carrying bacteria (*E. coli* and *Salmonella*) loaded with Doxorubicin for the targeting of tumoral cells [113]. The loading of liposomes in bacteria was done by the method of incubation and subsequent bacteria electroporation. This method allowed the engulfment of drug-loaded liposomes within bacteria, avoiding their fusion with the external membrane of bacteria and maintaining their integrity. They assessed the bacterial viability after engulfment of charged liposomes, observing that anionic liposomes only affected viability in a 20%, comparing with cationic liposomes that exhibited a 50% mortality. They also demonstrated that there was no significant reduction in the swimming capacity of liposome-loaded bacteria. Loaded bacteria were able to penetrate the tumor center at a faster rate than liposomes without bacteria, showing an enhanced tumor-killing effect. The therapeutic effect was provided by metabolic switch of Doxorubicin that release from bacteria once reached the tumor.

Dogra et al. took advantage of the high motility of *E. coli* bacteria to transport small, large, and giant unilamellar vesicles (SUVs, LUVs, and GUVs) [156]. For this, they utilized the inherent property of bacteria to bind with gangliosides (glycolipids) by incorporating glycolipids during vesicles synthesis. They found that bacteria could successfully propel SUVs and LUVs, observing a decrease in bacterial velocity as the vesicles become larger. However, due to their size, GUVs could not be propelled by attached bacteria.

Other example employing magneto-aerotactic bacteria, in this case *Magnetococcus marinus* strain MC-1, are the works reported by Felfoul et al. They employed this bacterium for magnetic guidance of drug-loaded nanoliposomes to hypoxic regions of HCT116 colorectal tumor xenografts [157]. Liposomes loaded with SN-38 antineoplastic drug were anchored to the surface of bacteria by carbodiimide coupling between carboxylic acid groups from liposome lipids and amine groups intrinsic to bacteria cell surface. They achieved the anchoring of approximately 70 drug-loaded nanoliposomes to each nanocarrier, and efficiency up to 55% of MC-1 nanosystem penetration into hypoxic regions of colorectal xenografts.

Here in Table 2, we present an overview of many of the existing bacteria-based nanobiohybrid systems with potential applications in cancer therapy.

## 6. Conclusions and Future Perspectives

The pathophysiology of solid tumors presents major problems in achieving good therapeutic outcomes with conventional treatments such as chemotherapy. On the contrary, experiments have shown that bacteria-based therapies can successfully promote tumor regression and promote survival in mice. Bacterial strains used for cancer therapy have unique characteristics, such as selectivity to tumors, unlimited gene delivery capacity, and the ability to proliferate in oxygen-deficient areas of tumors, which makes them good nanocarriers for targeted release of therapeutic agents in the inner regions of tumors. The works described in this review shows that the potential use of genetically modified facultative anaerobic bacteria, such as *Salmonella* or *E. coli*, among others, represents a good alternative to solve the poor penetration of chemotherapeutic agents in tumors. Thanks to motility and selective accumulation of these types of bacteria in tumor tissues, they become a powerful tool for the transport of drug-loaded nanomaterials, thus allowing a higher exposition of tumoral cells to therapeutic agents. These properties are enhanced by their ability to induce an innate response in the immune system from the host. Based on the characteristics described in this review, BMTT is expected to be an unbeatable candidate in the fight against cancer. The wide therapeutic toolbox that supposes genetically engineered bacteria can be exploited, in combination with new clinical strategies, and can transform cancer treatments into something more effective and safer.

## Figures and Tables

**Table 1:** Most common reactions for bioconjugation in living systems.

	Chemical Group (R_1_ or R_2_)	Partner Group (R_2_ or R_1_)		Conjugation Product		Ref.
**A**	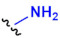	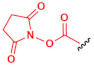	**NHS-ester**	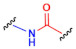	**Amide derivate**	[117]
**B**	**Amine**	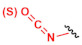	**Isocyanate or isothiocyanate**	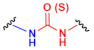	**(Thio)Urea derivate**	
**C**	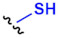	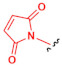	**Maleimide**	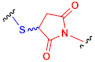	**Thioether derivate**	[117]
**D**	**Thiol**	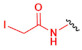	**Iodoacetamide**	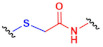	**Thioether derivate**	
**E**	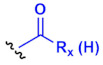 **Ketone or aldehyde**	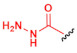	**Hydrazide**	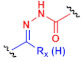	**Hydrazone derivate**	[114,117]
**F**		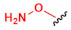	**Aminooxy**	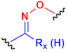	**Oxime derivate**	
**G**		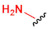	**Amine**	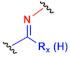	**Imine derivate**	
**H**	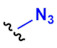 **Azide**	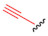	**Alkyne**	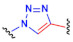	**Triazole derivate**	[114,115]
**I**		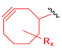	**Cyclooctyne**	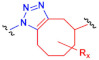	**Triazole derivate**	
**J**		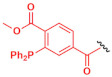	**Staudinger phosphine**	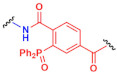	**Amide derivate**	

**Table 2:** Summary of bacteria-based biohybrid nanocarriers for cancer therapy.

Bacteria Type	NP-Bacteria Interaction	Bioconjugation Method	Nanomaterial	Therapeutic Strategy	Ref.
*L. monocytogenes*	Attached	Antigen/antibody and Avidin/neutravidin	Polystyrene NPs	Gene delivery and protein expression in tumoral cells	[97]
*E. coli*	Attached	Acid-labile linker	Free drug	Sustained release of drug	[136]
*Salmonella*	Adsorbed	Electrostatic interactions	PEI NPs	Cancer immunotherapy	[106]
*E. coli*	Attached	Tetrazine/norbornene click reaction	Polymeric pro-micelles	On-demand release of two drugs	[109]
*Salmonella*	Attached	Biotin/Streptavidin	PLGA NPs	-	[110]
*Magnetospirillum magneticum*	Attached	Michael addition to maleimide	Indocyanine green PLGA NPs	Photothermal therapy	[138]
*Salmonella*	Attached	Oxidation and self-polymerization	Polydopamine NPs	Photothermal therapy	[140]
*E. coli*	Adsorbed	Electrostatic interactions	Polyelectrolyte multilayer microparticles	Drug delivery with magnetic guidance	[141]
*E. coli*	Attached	Azide/DBCO click chemistry	MSNs	Transport of high amounts of drug	[112]
*E. coli*	Adsorbed	Electrostatic interactions	Carbon nitride NPs	Photoinduced in situ generation of cytotoxic species	[107]
*Clostridium novyi-NT spores*	Adsorbed	Electrostatic interactions	Branched Au NPs	Theragnostic combination therapy	[151]
*Bifidobacterium* and *Clostridium difficile*	Adsorbed/Attached	Electrostatic interactions and antigen/antibody	Au nanorods	Photothermal ablation	[152]
*E. coli*	Adsorbed	Metal-peptide affinity	Au NPs	-	[153]
*E. coli*	Attached	Carbodiimide chemistry	Fe_3_O_4_ NPs	Chemodynamic therapy	[154]
*E. coli* and *Salmonella*	Engulfed	Incubation and electroporation	Liposomes	Enhanced drug delivery	[113]
*E. coli*	Attached	Bacterial affinity with glycolipids	SUVs, LUVs, and GUVs	-	[156]
*Magnetococcus marinus*	Attached	Carbodiimide chemistry	Liposomes	Enhanced drug delivery	[157]

## Data Availability

The data presented in this work are available on request from the corresponding author.
